# Investigating target refraction advice provided to cataract surgery patients by UK optometrists and ophthalmologists

**DOI:** 10.1111/opo.12957

**Published:** 2022-02-18

**Authors:** Emily Charlesworth, Alison J Alderson, Fiona Fylan, Richard A Armstrong, Aman Chandra, David B Elliott

**Affiliations:** ^1^ Bradford School of Optometry and Vision Science University of Bradford Bradford UK; ^2^ Leeds Sustainability Institute Leeds Beckett University Leeds UK; ^3^ School of Optometry College of Health and Life Sciences Aston University Birmingham UK; ^4^ Vision and Eye Research Institute Anglia Ruskin University Cambridge UK

**Keywords:** cataract surgery, health decision making, spectacles, target refraction

## Abstract

**Purpose:**

To determine whether UK optometrists and ophthalmologists provide target refraction advice to patients prior to cataract surgery, and when this should first be discussed.

**Methods:**

Optometrists and ophthalmologists were asked to complete a survey of two clinical vignettes (both older patients with cataract; a pre‐operative myope who routinely read without glasses and a patient using a monovision approach), plus multiple choice and short answer questions either using hard copy or online.

**Results:**

Responses were obtained from 437 optometrists and 50 ophthalmologists. Optometrists who reported they would provide target refraction advice were more experienced (median 22 years) than those who would leave this to the Hospital Eye Service (median 10 years). The former group reported it was in the patients’ best interest to make an informed decision as they had seen many myopic patients who read uncorrected pre‐operatively, and were unhappy that they could no longer do so after surgery. Inexperienced optometrists reported that they did not want to overstep their authority and left the decision to the ophthalmologist. The ophthalmologists estimated their percentage of emmetropic target refractions over the last year to have been 90%.

**Conclusion:**

Currently, some long‐term myopes become dissatisfied after cataract surgery due to an emmetropic target refraction that leaves them unable to read without glasses as they did prior to surgery. Although experienced optometrists are aware of this and attempt to discuss this issue with patients, less experienced optometrists tend not to. This suggests that target refraction needs greater exposure in university training and continuing professional development. To provide patients with the knowledge to make informed decisions regarding their surgery, we suggest an agreed protocol within funded direct referral schemes of initial target refraction discussions by optometrists to introduce the idea of refractive outcomes and outline options, with further discussion with the ophthalmologist to clarify understanding.

**Key points**

- Many experienced optometrists reported long‐term myopic patients were dissatisfied with a target refraction of emmetropia due to the need to wear reading glasses following cataract surgery.
- Less experienced optometrists discussed target refraction much less with cataract surgery patients, suggesting this needs further exposure in university education and continuing professional development.
- The inclusion of initial target refraction discussions by optometrists within funded direct referral schemes seem to be a useful step forward.

## INTRODUCTION

Cataract surgery is a commonly performed procedure, with an estimated 25 million cataract operations performed each year worldwide.^1^ Advancements have not only led to an increased number of patients undergoing cataract surgery, but also an increase in patients’ expectations from surgery.^2^ Post‐operative satisfaction has been evaluated in a variety of ways including quality of life scores,^3^, ^4^ subjective satisfaction^5^, ^6^ and the ability to perform vision dependent tasks.^7^ Fewer studies have looked at the refractive expectations of patients following surgery.^8^ While the main aim of cataract surgery is to improve vision by replacing the opacified lens with a clear intraocular lens (IOL), surgery is increasingly recognised as a refractive procedure, with the power of the IOL inserted during cataract surgery calculated to achieve a target refraction. The power of the IOL involves accurate keratometry, biometry and anterior chamber depth measurements, plus the selection of an appropriate IOL power formula.^9^, ^10^ Obtaining the correct target refraction is needed to ensure visual needs are met and the patient is happy with the outcome of surgery.^11^ Patients in the UK currently receiving surgery under the publicly funded National Health Service (NHS) are only eligible for monofocal IOLs, and so will require distance or near vision spectacles for complete visual restoration following surgery. Despite this, it has been demonstrated that a main motivation to undergo surgery is to achieve spectacle independence.^8^ More important still is that perhaps patients who did not require spectacles before surgery for distance and/or near do not expect to require them afterwards.^8^ It is therefore important to determine and manage patients’ expectations of refractive outcomes.

A focus group study exploring patients’ experiences of their vision following cataract surgery found a lack of knowledge among patients of how their final prescription would impact the type of spectacles worn post‐surgery.^11^ This study, and others^12^, ^13^ showed that some patients who were myopic pre‐surgery and preferred to read without spectacles struggled with their post‐surgical emmetropia, and would have preferred a myopic target refraction to allow them to continue to read without spectacles post‐surgery.^12^, ^13^ Similarly, it has been shown that patients who read without spectacles before cataract surgery would expect to be able to read without them after surgery.^8^ Such older patients would be low myopes with relatively little astigmatism, and because they would wear distance spectacles before surgery, they would expect to also wear them afterwards.^8^ To ensure patient satisfaction and minimise refractive disappointment, patients should have a thorough discussion regarding their refractive expectations and spectacle use post‐operatively.

This is an exploratory study using a survey sent to UK optometrists and ophthalmologists and a mixture of vignette case scenarios, plus multiple choice and short answer questions. The aims of the study were:

1. To determine if optometrists and ophthalmologists provide advice regarding post‐operative target refraction.
2. To determine the optimum time target refraction should first be discussed with the patient.

As previous studies have shown differences in optometric referrals and NHS sight test outcomes depending on optometrist experience and practice type (typically large groups of optical practices (multiple) versus independents),^14^, ^15^ these factors were explored in this study.

## METHODS

### Overview

Ethical approval was gained from the Chair of the Biomedical, Natural, Physical and Health Sciences Research Ethics Panel at the University of Bradford on 08/04/2020 (EC26122). The survey gathered background information regarding the number of years qualified, the work setting (large multiple, small multiple up to 10 branches, independent, hospital, university eye clinic or other for optometrists; NHS hospital, private practice or both for ophthalmologists) and resident (i.e., permanent appointment) or locum practitioner status for optometrists. It was then split into two sections: the first consisted of two vignette clinical case scenarios (Patients A and B) and the second consisted of follow up questions regarding when target refraction should be discussed, and what percentage of patients receive an emmetropic target refraction (see Appendix 1). The survey included free text questions to give practitioners the opportunity to explain their answers. The number of questions were kept to a minimum to allow the survey to be completed in approximately 10 min in order to gain a good response rate.

The optometrist years qualified data were described using non‐parametric statistics, as the data were shown not to be normally distributed by Kolmogorov‐Smirnov tests. A Fisher's Exact test was used to compare target refraction discussion rates among large multiple and independent practice types. A multinomial logistic regression was used to assess if any of the three variables (i) practice type, (ii) years qualified or (iii) resident/locum were associated with discussing target refraction. First, a main effects model was used to assess the effect of each independent variable upon the answer chosen. Then, a full factorial model was conducted to investigate interactions between the independent variables. The ophthalmologist years qualified data were described using parametric statistics, as the data followed a normal distribution.

### Part A – Case scenarios

The vignette case scenarios were agreed by three optometrists (authors EC, AJA and DBE, 2–36 years qualified) and an ophthalmologist (author AC, 19 years qualified). The scenarios were identical for both the ophthalmologist and optometrist questionnaires, but the questions surrounding the scenario were different to fit the role of each profession. The scenarios were written to assess a key theme identified in the focus group study by Webber et al.^11^ This investigated whether practitioners would discuss target refraction with the patient before surgery (optometrists at the point of referral, ophthalmologists pre‐operatively) and how this would affect their long‐term post‐operative correction following surgery. Ophthalmologists were also asked what target refraction they would recommend.

Patient A and Patient B both had bilateral cataracts. Patient A was a pre‐operative myope (−2.50DS) who wore distance single vision spectacles for everyday wear, taking the spectacles off to read. This prescription was chosen as it allowed the patient to be able to read comfortably without their spectacles and addresses the issue of some myopic patients struggling with post‐surgical emmetropia.^12^, ^13^ Patient B habitually used a monovision contact lens approach, with their right eye set for distance vision, and their left eye set for near, and expressed that spectacle independence was important. A pilot version of the questionnaire received comments from 10 optometrists and one ophthalmologist and lead to some minor wording revisions.

### Part B – Further questions

Part B consisted of further questions and asked when practitioners thought a patient's target refraction should be discussed. The options were: (i) by the optometrist at the point of referral (direct referral), (ii) by the optometrist at the point of referral (GP referral) and (iii) by the ophthalmologist. Ophthalmologists were asked one additional question, investigating the percentage of patients in which they aimed for an emmetropic target refraction in the last year.

### Distribution

#### Optometrists

Postal and online recruitment methods were used from the start of the study in an attempt to recruit as many optometrists as possible. The questionnaires were distributed between December 2019 and March 2020. A prospective sample size of 411 was determined following the method of Israel^16^: a population size of 14,000, confidence interval of 95% and margin of error of 5% resulted in a sample size of 374. We then added an extra 10% for missing data or responses needing to be discarded. A random number generator was used to select optometrists from the UK General Optical Council register. An information sheet, a copy of the questionnaire and a free return envelope was sent to the practitioners’ registered addresses. A total of 235 questionnaires were sent out. An online version of the questionnaire was also created using Google forms. The link to the form was distributed on social media platforms, forums and to The College of Optometrists. All 75 local optical committees in the UK were contacted to ask if they were willing to distribute the questionnaire to the practitioners in their area. The link was also distributed to all universities providing an undergraduate optometry degree.

#### Ophthalmologists

The questionnaires were distributed between August 2020 and December 2020. A prospective sample size of 326 was determined following the method of Israel.^16^ A population size of 1300, confidence interval of 95%, and margin of error of 5% resulted in a sample size of 297. We then added an extra 10% for missing data or responses needing to be discarded. The survey was distributed to ophthalmologists within the UK using email addresses obtained from the Royal College of Ophthalmologists’ website. A link to the survey was also shared on social media platforms, and further distributed by author AC and other ophthalmologists who contacted us offering to assist with the distribution to increase response rates.

## RESULTS

### Optometrists’ responses

A total of 437 questionnaires were returned consisting of 359 online responses and 78 paper replies (a response rate of 33%). 78.5% of the responses (343) were from resident optometrists and 21.5% (94) from locum optometrists. Table 1 shows the responses per practice type and the corresponding median number of years qualified. Optometrists from large multiples were significantly less experienced (22% had less than 3 years’ experience) compared to optometrists from independent practices (4%) (U = 7142, *p *< 0.001).

**TABLE 1:** The number of years qualified for 437 UK optometrists completing the survey divided by practice type

Type of practice	% of total 437 questionnaires returned	Median (IQR) years qualified
Large multiple	195 (45%)	10 (5–20)
Independent	169 (39%)	25 (17–35)
Hospital	40 (9%)	15 (8–22)
Small Multiple (less than 10 branches)	18 (4%)	21 (15–27)
University	9 (2%)	26 (8–30)
Domiciliary	6 (1%)	21 (15–29)

### Patient A

Patient A was a simple myope of −2.50DS in both eyes who took their spectacles off to read. Optometrists were asked, “At the point of referral, would you discuss with the patient their target refraction options? (e.g., an emmetropic target refraction and distance spectacle independence, a myopic target refraction and near spectacle independence or an monovision target refraction and spectacle independence)”. Three options were available: (i) yes, discuss in person (34% of responses), (ii) yes, and state preference in referral letter (28% of responses) or (iii) no, leave the patient to discuss this with the ophthalmologist/ hospital eye service (38% of responses). Responses to Patient A were further divided into practice type and can be seen in Table 2.

**TABLE 2:** Table showing the number of survey responses for Patient A in each category

	Yes, discuss in person	Yes, and state preference in referral letter	No, leave the patient to discuss this with the ophthalmologist/HES
	*n* = 149	*n* = 123	*n* = 165
	Median years qualified (IQR): 20 (10–30)	Median years qualified (IQR): 22 (16–31)	Median years qualified (IQR) 10 (15–20)
Large multiple *n* = 195	54 (28%)	31 (16%)	110 (56%)
Independent *n* = 169	72 (43%)	63 (37%)	34 (20%)
Small multiple *n* = 18	5 (28%)	8 (44%)	5 (18%)
Hospital *n* = 40	11 (28%)	16 (40%)	13 (33%)
Domiciliary *n* = 6	0 (0%)	4 (67%)	2 (33%)
University *n* = 9	7 (78%)	1 (11%)	1 (11%)

Each category is further subdivided into responses per practice type. HES, Hospital Eye Service.

When comparing results from practice type, four options included small amounts of data (<10%) and were considered too small for meaningful analysis. We compared data from the two largest groups of large multiple (N = 195) and independent (N = 169). The results showed highly significant differences (Table 2), with “Discuss in person”: 28% large multiple vs. 43% independent; “Discuss and state in referral letter”: 16% large multiple vs. 37% independent; “leave discussion to HES”: 56% large multiple, 20% independent (Fisher's Exact test, *p *< 0.0001). Multinomial logistic regression found a statistically significant association between discussing target refraction and years qualified (χ^2^(2) = 21.2 *p *< 0.0001), with a non‐significant interaction between practice type and whether the optometrist was a resident/locum, (χ^2^(2) = 0.47 *p *= 0.80). This suggests that differences were due to years of experience and not practice type. Binary logistic regression produced an odds ratio of 1.07 (95% CI 1.05–1.09) indicating a 6.6% increase in discussion rates per year of experience.

### Patient B

Patient B was a habitual monovision contact lens wearer with his right eye for distance vision and his left for near vision and spectacle independence was important to them. The importance of spectacle independence made the situation unique, potentially making it more obvious to the respondent that they should discuss target refraction with the patient. Optometrists were asked the same question, “At the point of referral would you discuss with the patient their target refraction options? (e.g., an emmetropic target refraction and distance spectacle independence, a myopic target refraction and near spectacle independence, or an monovision target refraction and spectacle independence)”. The same three options were available: (i) yes, discuss in person compromising (26% of responses), (ii) yes, and state preference in referral letter (46% of responses), and (iii) no, leave the patient to discuss this with the ophthalmologist/ hospital eye service (28% of responses). Responses to Patient B were further divided into practice type and can be seen in Table 3.

**TABLE 3:** Table showing the number of survey responses for Patient B in each category

	Yes, discuss in person	Yes, and state preference in referral letter	No, leave the patient to discuss this with the ophthalmologist/HES
	*n* = 113	*n* = 202	*n* = 122
	Median years qualified (IQR): 20 (10–30)	Median years qualified (IQR): 20 (11–30)	Median years qualified (IQR) 10 (5–20)
Large multiple *n* = 195	48 (25%)	68 (35%)	79 (40%)
Independent *n* = 169	49 (29%)	94 (56%)	26 (15%)
Small multiple *n* = 18	4 (22%)	11 (61%)	3 (17%)
Hospital *n* = 40	7 (28%)	21 (53%)	12 (30%)
Domiciliary *n* = 6	0 (0%)	4 (67%)	2 (33%)
University *n* = 9	5 (56%)	4 (44%)	0 (0%)

Each category is further subdivided into responses per practice type. HES, Hospital Eye Service.

A Fisher's Exact test showed that the distribution of those who would discuss or not discuss target refraction with Patient B were significantly different from each other in large multiple and independent practices (*p *< 0.0001). A full factorial, multinomial logistic regression showed a statistically significant association between discussing target refraction and years qualified (χ^2^(2) = 11.9 *p *= 0.003), with a non‐significant interaction between practice type and whether the optometrist was a resident/locum, (χ^2^(2) = 0.30 *p *= 0.86). This suggests that differences were due to years of experience and not practice type.

Further analysis was conducted to compare the responses optometrists gave for both Patient A and Patient B. They were subdivided into those that discussed target refraction with both patients, those that did not discuss target refraction with either patient or those that would discuss target refraction with only one of the patients (Table 4). There was a significant difference in the years qualified between the four groups (Kruskal Wallis test H_3_ = 52.0, *p* < 0.0001.

**TABLE 4:** Optometrists’ median years qualified when subdivided into those that discussed target refraction with both patients, with neither patient or with only one of the patients

	*n*	Median (IQR) years qualified
Discuss with both Patient A and B	265	22 (13–31)
Do not discuss with Patient A or B	114	10 (5–20)
Discuss with Patient B only	50	15 (5–20)
Discuss with Patient A only	8	14 (10–20)

### Qualitative analysis

**FIGURE 1:**
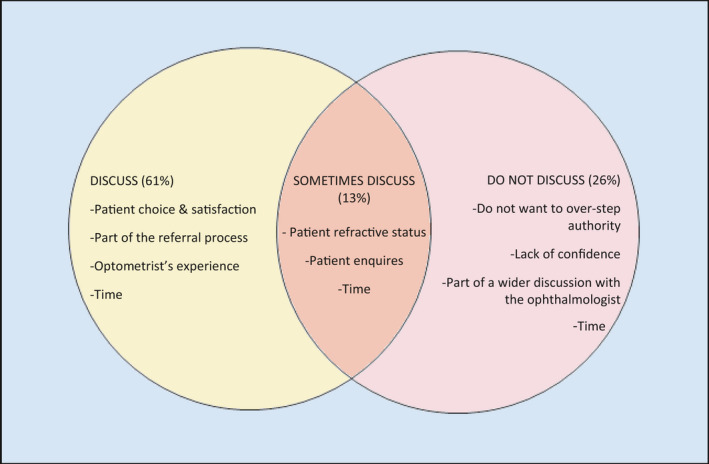
Venn diagram showing the themes found during qualitative analysis, and the percentage of comments found in each theme

Practitioners were also asked to explain their responses, and these were analysed using inductive content analysis whereby themes are generated from the data set.^17^ Two coders (EC and FF) were used to code and organise the data into categories using as many headings as necessary to fully describe the content. The list of categories were then grouped with other similar categories to create themes. We aimed to identify any common themes within responses of those that reported they would: (i) discuss target refraction, (ii) would not discuss it and (iii) would discuss it occasionally. The comments from each group provided themes that helped to explain their decision (Figure 1). These are described below and demonstrated with example quotes. Time was an overarching theme and found within comments from all groups.

### Optometrists who discuss target refraction

The reasons why optometrists reported they would discuss target refraction could be explained by three themes.

1. Patient choice and satisfaction

This was the most common theme found. Optometrists highlighted the importance of giving patients a choice of their target refraction to ensure that their vision is optimal for their lifestyle, and to help ensure they will be satisfied with the results of surgery. Many emphasised the importance of explaining the options to patients, so they have a better understanding of the impact of their decision and can make an informed choice. Several noted the importance of giving patients the chance to think about their options before seeing the ophthalmologist to prevent them from making a snap decision without fully understanding or considering the implications. A few also noted that the patient is given a lot of information at their hospital appointment, which may mean that they do not digest or understand all the options, and therefore reduces their ability to make a considered decision about target refraction. Accordingly, discussing this earlier may lead to a better patient outcome.

Very important to discuss as refractive outcome massively affects quality of life post op and patients perception of success of the op.‐ Respondent 157

I'm primarily a hospital optom for a cataract surgery provider, so always discuss the refractive target at pre‐assessment in secondary care. The number of myopic patients that this choice catches off guard is significant. When in a primary care setting, I'd mention the options to plant the seed of them thinking about what they want before they reach the hospital, and leave them time to make the decision. – Respondent 81

1. Optometrists’ experience of unhappy patients

Many optometrists reported a history of examining patients dissatisfied with the refractive outcome of cataract surgery, and this was their main motivation in discussing target refractions. Some felt patients may not be given a choice about their target refraction as all options may not be offered, or it may not be explained in a way the patient understands. In particular, some optometrists worried that some (particularly myopic) patients could accept an emmetropic target refraction without fully understanding its consequences on their visual needs and lifestyle.

A lot of them come back post cataract and say they feel worse off because they could see for near but after surgery they can no longer see. It's best to already ask the patient their preference or talk about it so they can make up their mind by the time they are seen at HES (hospital eye service) – Respondent 52

I have had too many myopes disappointed with their emmetropia (and therefore need for readers!) and know that I too would prefer to be left myopic, so always try to inform the patient at this point. – Respondent 335

1. Part of the referral process

Some optometrists reported that discussing target refraction was part of their referral process. This included optometrists that felt they had a duty of care to discuss options and outcomes of surgery with patients, and those where this was part of a funded cataract referral pathway. Many felt they were in a good position to discuss and advise patients on this topic as they have a good understanding of patients’ refractive history and visual needs.

It’s important, and necessary, I believe, our responsibility as a primary care professional to discuss different possible outcomes with the patient and discuss patient preferences. I also think it’s important to pass this information to the ophthalmologist in writing.‐ Respondent 117

We have a funded extended counselling service for cataract referral and this would be a part of it. – Respondent 30

### Optometrists who do not discuss target refraction

The reasons why optometrists who reported they would not discuss target refraction, did not do so could be explained by three themes.

1. Do not want to over‐step authority

Some optometrists felt that discussing target refractions with a patient could be over‐stepping their authority.

I am not operating or choosing the lens power of the implant. It may be different if a long term monovision contact lens wearer but I would not patronise the surgeon into telling them which refractive result to aim for in this case. – Respondent 136

I do not make a recommendation at the risk of stepping on ophthalmologist’s toes. – Respondent 268

1. Lack of confidence

Some felt a lack of confidence in what options were available, and others were unsure if the ophthalmologist had any specific preferences. Some highlighted that they would not feel confident to have the discussion in practice, as there may be clinical factors they are not aware of that may limit target refraction. A few described how having this discussion with patients could lead to disappointment if a patient chooses a target refraction that the ophthalmologist later decides is not an option. Others lacked confidence that their recommendations would be seen by the ophthalmologist or would be ignored.

Would not feel confident offering the patient other options when I am not sure that the ophthalmologist is happy to agree to it. – Respondent 21

I wouldn't want to create expectations that were then changed @ HES for a reason. Also not confident in discussing what could be achieved. – Respondent 347

1. Ophthalmologist role

This included all responses believing that the responsibility to discuss the target refraction lies with the ophthalmologist rather than themselves (optometrists). Many talked about how the ophthalmologist makes clinical decisions about which target refraction to offer, and extra examinations and tests are often conducted at the hospital before a target refraction discussion can occur. Some thought that the discussion about target refraction best takes place alongside a wider discussion of the risks of surgery and what the patient will experience. Some described how it had never occurred to them that they would have this discussion with the patient, as everything to do with surgery is handled by the hospital. Several optometrists noted that they did not discuss target refraction with the patient as it is *not* required as part of the referral process. This reinforces the idea that a discussion is not part of the optometrist's professional role and will be covered by the ophthalmologist.

This decision needs to be made between the ophthalmologist and the patient. We can refer but ultimately, it's the surgeon's decision whether he performs surgery. It's their responsibility to discuss the pros, cons and risks of the procedure and the potential outcome for the patient. – Respondent 210

It is down to what the surgeon thinks is suitable for the patient as they will do further tests to determine what is the correct IOL power, axial length, etc which we as optometrist cannot do so. – Respondent 124

### Optometrists who sometimes discuss target refraction

Optometrists who sometimes reported that they would discuss target refraction said they would do so for the monovision patient but were less likely to do so for the myopic patients, and that they would also provide information if, and when, asked.

### Time

Time was an encompassing theme for all three groups. In those that discussed target refraction with patients, they did so because they felt they had the time to discuss different options with the patient. Some felt they had more time than would be available to the ophthalmologist at the hospital, and that ophthalmologists may not spend sufficient time on the discussion due to time limitations. One hospital optometrist stated there was not enough time to discuss target refraction options at the hospital, and some mentioned that local ophthalmologists requested that optometrists have this discussion with patients, further demonstrating that the time available during hospital appointments was limited.

Not often time to discuss fully in hospital, and good that the patient is fully aware of their options – Hospital optometrist – Respondent 96

Local ophthalmologists have requested this as it means their time is better spent with the patient if options have been discussed – Respondent 25

Of those that do not discuss target refraction, many mentioned this was due to time limitations in practice.

Time constraints in practice do not allow enough time to spend with patients to go through different correction options. – Respondent 135

We have no time to discuss further options due to a 25‐minute slot to complete an eye‐test – Respondent 124

### Sometimes discuss

Of those optometrists who would only discuss target refraction sometimes, time availability was one of the main contributing factors. They noted they would only discuss target refraction with the patient if they had time but if not, they would advise the patient to discuss this when they saw their ophthalmologist. Some felt they did not have sufficient time to have an in‐depth discussion, but would briefly mention the options available if time allowed.

Will only discuss if I'm running on time. If not then I'll briefly mention that it's something to talk about with your consultant. – Respondent 159

Time issue ‐ have discussed with patients when he/she wants more information. – Respondent 318

#### Ophthalmologists’ responses

A total of 50 questionnaires were completed, of which all were completed online. Ophthalmologists working in the NHS completed 54% of responses, 6% worked in private practice, and 40% worked a combination of NHS and private practice. The average number of years qualified was 18.3 years (SD 7.3 years). The sample size is much lower than our target number of 326, and subgroup sample sizes (e.g., practice type, years of experience) are too small for meaningful analyses. Table 5 shows the responses for the myopic Patient A and Table 6 for the monovision Patient B.

**TABLE 5:** Survey responses of 50 ophthalmologists for Patient A and Patient B

Patient A Would you discuss with Patient A their target refraction options before surgery? Please state your reason why.	%	Patient B Would you consider a monovision target refraction for Patient B and state your reasons why.	%
Usually recommend an emmetropic target refraction	24	Yes, usually recommend a monovision target refraction	52
Usually recommend a myopic target refraction	18	No, usually recommend an emmetropic target refraction	6
Accept the patient's decision following a discussion of available options with the patient	52	No, usually recommend a myopic target refraction	2
Accept the patient's decision based on the discussion they had with their optometrist	6	Accept the patient's decision following a discussion of available options with the patient	40
No discussion	0		

Ophthalmologists were asked over the past year what percentage of patients they aimed for an emmetropic target refraction. The median response was 90%, range 80%–100%.

### Ophthalmologists who discuss target refraction

Many ophthalmologists highlighted the importance of discussing target refraction with the patient to ensure they are satisfied with the outcome of surgery, and saw this as best practice.

Important to explain options to the patient and allow own decision based on their visual requirements and preferences … in my experience not all low myopes are happy with emmetropia – Respondent 30

Would not make decision based on what patient had discussed with their optometrist. Would have discussion with the patient and together we would make the decision. Nine times out of ten, emmetropia is chosen – Respondent 17

#### Ophthalmologists who discuss and make recommendation

This category covers ophthalmologists who would discuss available options with the patient and then recommend a target refraction. The target refraction advised was for several reasons: some advised a specific target refraction due to the patient's lifestyle or hobbies, and some based on their past clinical experience. Several suggested discussing all options with the patient, and then trialling different refractive errors with contact lenses to ensure the patient is happy with their choice before making a recommendation. This latter approach seems problematic as although post‐operative emmetropia with ready‐made readers for near might be easily simulated, post‐operative myopia would also require new distance spectacles that they would remove for near work.

I tend to say that we usually aim for emmetropia but offer myopia if they wish ‐ they tend to go for emmetropia – Respondent 26

The refractive aim will depend on the patient’s daily activities, e.g., some myopes read unaided, and may want to continue to do this. If they are unsure I would suggest a contact lens trial to simulate the outcome, informing them of the degree of prediction error in outcomes. – Respondent 26

### Ophthalmologists who make recommendations only

This category covered ophthalmologists who make a target refraction recommendation for the patient but did not mention discussing options. Of those that recommended a target refraction for the myopic Patient A, 57% reported that they would recommend an emmetropic target (although several suggested a low myopic target of around −0.75DS and reading glasses). Most of the responses were based upon clinical experience and satisfaction of previous patients.

Normally aim at ‐0.75 to ‐1.0 as myopes ‘hate’ ending up on hypermetropic side of emmetropia – Respondent 15

Low myopia and reading glasses are acceptable for most patients. Taking off glasses to read appears to be a new practice for this teacher. If they are clear about their needs and have had a robust discussion with their optom and is documented in the referral letter then I would go with, ‘usually accept the patients decision based on the discussion they had with their optometrist‐ Respondent 11

**FIGURE 2:**
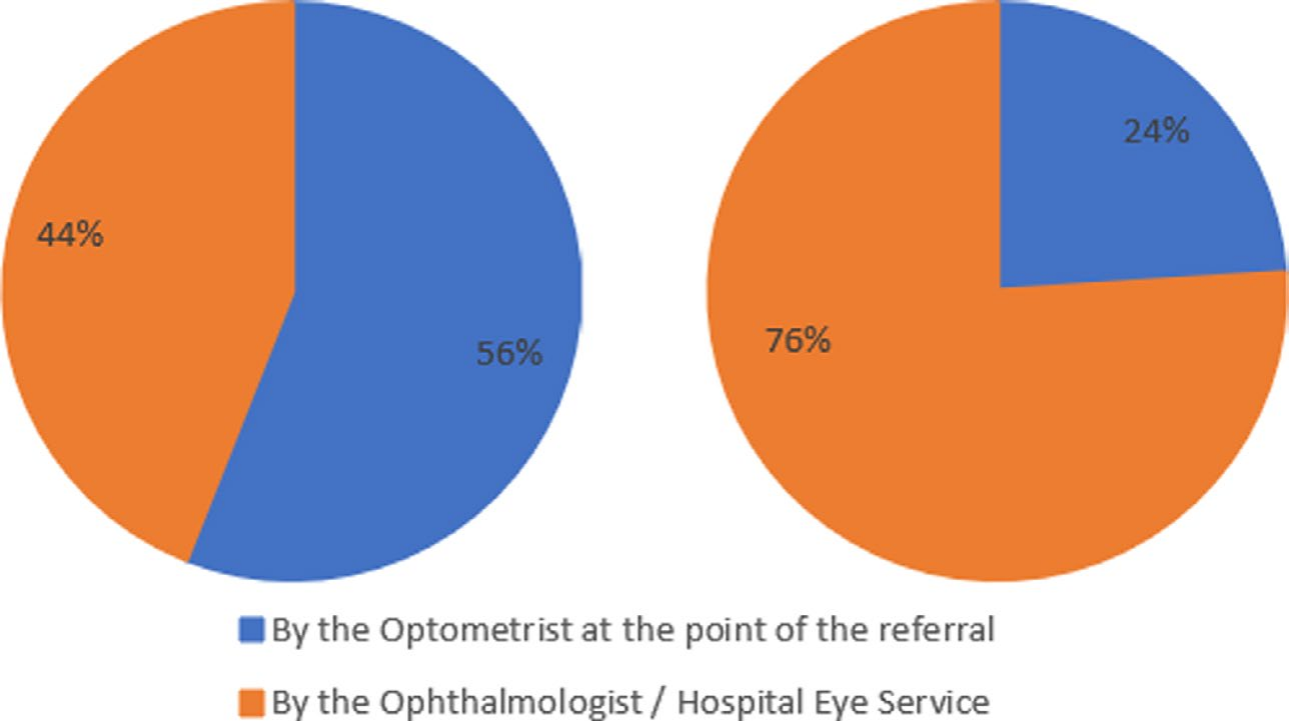
Showing the responses from optometrists (left) and ophthalmologists (right) when asked when target refraction should first be discussed with the patient

Finally, both optometrists and ophthalmologists were asked when they thought target refraction should first be discussed with the patient, with the results shown in Figure 2.

### Managing expectations

When discussing target refraction with patients, it is important that the patient's expectations are managed. While the patient has a choice when deciding upon their final target refraction, they should be made aware that this is within a tolerance and cannot always be guaranteed. National biometry audits suggest a benchmark of 85%–90% of patients undergoing routine cataract surgery should achieve a final spherical equivalent refraction within 1 dioptre of the predicted value, and 55% of patients should be within 0.5 dioptres.^18^, ^19^ It should be emphasised the main aim of surgery is to remove the cataract rather than correct for any refractive error. Indeed, this issue was raised by one optometrist working within the Hospital Eye Service who discussed how over promising results can be problematic and is not always fully discussed with the patient.

I currently work for an AQP (Any Qualified Provider – An NHS contract which allows non NHS organisations to provide NHS services) doing cataract surgery. Community optometrists discussing refractive targets is a big problem we face. The reason for this is because community optometrists don't tend to explain that the reason for cataract surgery is to improve vision whether they need specs for distance vision and near vision post operatively or not. The patient doesn't understand the ±1.00 tolerance of the operation (because it is often not explained) and when we try and discuss with the patient a number of them are upset and say, 'Well my optometrist said … '. Not only does this make the Community optometrist look like they don't know what they are doing it leaves patients very unhappy.‐ Respondent 196

## DISCUSSION

Target refraction discussions are vital to ensure patient satisfaction post‐operatively. Current advice from the NHS states, “Providers must ensure that patients have sufficient time to consider these complex issues, and decisions such as post‐operative target refraction should be determined and agreed with the patient well in advance of the scheduled surgery date.”^20^ Despite this, a recent focus group study (*n* = 26) found 27% of patients had no discussion regarding their target refraction or were not given sufficient time or information to make an informed choice, and therefore deferred to the ophthalmologist's recommendation.^11^ This study aimed to investigate if clinicians did discuss target refraction with their patients. We found both optometrists and ophthalmologists had differing views of when target refraction should first be discussed with the patient. Some optometrists were found to not discuss target refraction options at all with patients, while others would have a preliminary discussion. Likewise, some ophthalmologists preferred to recommend a target refraction (typically emmetropia) while others had a discussion of available options before accepting the patient's decision.

Optometrists and ophthalmologists participating in our survey were found to have more agreement for the monovision patient (Patient B) and typically felt that a full discussion and possibly contact lens trials were warranted for this patient. The pre‐operative low myope (Patient A) provided more differing views. Less experienced optometrists were found to not discuss target refraction for two reasons. Firstly, they were not familiar with previously myopic patients being unhappy with their final refraction (emmetropia) because they had now lost their ability to read without spectacles. Secondly, they reported a lack of confidence and knowledge and a desire not to over‐step their authority. The multinomial logistic regression results suggested that the reason that optometrists in large multiples discussed target refraction much less than those in independent practices is that these groups employ far more inexperienced clinicians. The driver appears to be years of experience and not practice type.

Only a small number of responses were received from ophthalmologists (50) and much lower than our target size (326), so the results cannot be generalised and must be treated with caution. The majority of ophthalmologists (52%, 26/50) reported they would discuss the available options with the myopic patient and then allow them to make an informed decision, while 42% (21/50) reported that they would recommend either emmetropia (12/50, 24%) or myopia (9/50, 18%), although the comments provided suggested that the latter was low myopia of about −0.75 to −1.00DS. Five out of the nine ophthalmologists who would recommend myopia would advocate low myopia, which would still require patients to use reading glasses rather than a higher myopic distance correction (≈ −2.00D) that would allow reading without glasses. Ophthalmologists reported targeting an emmetropic refraction in 90% of their patients in the past year, with the remaining 10% consisting of mainly myopic patients with some monovision target refractions. It is difficult to estimate how many myopic patients who read uncorrected are obtaining an emmetropic target refraction. In Europe, those born between 1940 and 1979 have been shown to have a myopic prevalence of 23.5% pre cataract surgery.^21^ The refractive error UK biobank study demonstrated a myopic prevalence of 24.9% and 20.1% in those 60–64 and 65–69 years of age, respectively. From our small sample estimate of at most 10% targeting a myopic refraction, this suggests that at least half of myopic patients may be obtaining an emmetropic target refraction post‐operatively.^22^ However, this is further complicated by astigmatism, which would need to be relatively small to allow reading uncorrected.

Target refraction discussions are essential for every patient, and our results highlighted several barriers to patients receiving these discussions. We discuss below how these barriers can potentially be overcome. Firstly, education. The 44% (191/437) of optometrists who believe target refraction should be discussed by the ophthalmologist were significantly less experienced (Fisher's Exact test *p *< 0.0001) and discussion rates were shown to increase 6.6% per year of experience. To improve the discussion rate in this group, including this topic in optometric education would raise awareness of the issues patients face post‐surgery, and improve confidence for the more inexperienced. Continued professional development could be provided for qualified optometrists.

Secondly, referral schemes. Funded direct cataract referral schemes exist in some areas of the UK, whereby optometrists are paid a fee to allow them to spend additional time counselling a patient about the pros and cons of cataract surgery before referring directly to ophthalmology. However, the main aim of these schemes is to reduce the number of patients referred who then decline treatment, rather than to introduce a target refraction discussion with the patient. Out of our 437 optometrists surveyed, only one specified it was part of their referral scheme. Many optometrists did not discuss target refractions as there was no option to add this onto the referral form, or they did not believe that the letter they sent would be available to the ophthalmologist when they saw the patient. Including target refraction as part of a direct referral or shared care scheme would encourage optometrists to discuss this with their patients.

Thirdly, time appears to be a significant barrier for optometrists discussing target refraction in practice. While a handful of optometrists felt they had time to discuss this and show available options to patients, many felt time constraints would not allow for a discussion. A funded direct referral scheme including the optometrist having an initial target refraction discussion with the patient is likely to free up more time for ophthalmologists who also raised limited time as an issue.

### Limitations

A limitation of the study is that we did not directly gather information regarding whether individual optometrists were involved in direct cataract referral schemes that included discussing target refraction as part of their referral process. Analysis of the ophthalmology data was limited by the small number of responses in this group. There are several explanations for the low response rate: (i) the time constraints of ophthalmologists, (ii) the changing of NHS email addresses making it harder for us to contact ophthalmologists and (iii) some ophthalmologists reporting NHS firewalls blocking our emails. The ophthalmic directory from the Royal College of Ophthalmologists was used to contact all of the ophthalmologists with an available email address, and they were asked to respond and circulate. An ophthalmologist was part of the study team (AC) and attempted to obtain responses from colleagues and several other ophthalmologists helped with circulations, along with promoting the study on social media platforms. Despite these attempts, the response rate remained low. Although the results should be interpreted with caution, we believe that the data are valuable in helping to demonstrate that the great majority of patients have an emmetropic target refraction (90%, range 80%–100%) and 12 of the 50 ophthalmologists (24%) reported that they would aim for a target refraction of emmetropia for the moderate myope who read without spectacles pre‐surgery. Finally, as the respondents were self‐selecting these clinicians may have had an interest in target refraction, and this may have influenced the results and introduced bias.

## CONCLUSION

Opinions differed between optometrists and ophthalmologists of when target refraction should first be discussed with patients when referring for cataract surgery. Experienced optometrists were much more likely to discuss target refraction with their patients (*p *< 0.0001), with less experienced optometrists choosing not to due to lack of experience, confidence and wishing to avoid patronising the ophthalmologist. We assume that the view of experienced optometrists, with their knowledge of the unhappy patients produced under the current system, is the approach to follow. This suggests that this issue needs greater exposure in university training and continuing professional development. Both optometrists and ophthalmologists raised time constraints as a barrier to having comprehensive discussions concerning target refraction. Funded direct referral schemes are likely to be a useful step forward so that optometrists are provided with the extra time to discuss this with patients, who will then be more informed for the discussion they will have with their ophthalmologist and will allow them sufficient time to consider their decision. Despite many optometrists already discussing target refraction options with patients, within our study, 76% of ophthalmology respondents thought target refraction should first be discussed by themselves. Guidelines developed between optometrists and ophthalmologists would both provide optometrists with the confidence and correct information of what to include in patient discussions, and provide confidence to ophthalmologists that the patient is correctly informed before they attend the Hospital Eye Service. An agreed protocol of initial target refraction discussions by optometrists to introduce the idea of refractive outcomes, and outline options with further discussion with the ophthalmologist to clarify understanding and make a decision, will help to provide patients with the knowledge and time to make informed decisions regarding their surgery.

## CONFLICTS OF INTEREST

The authors report no conflicts of interest and have no proprietary interest in any of the materials mentioned in this article.

## AUTHOR CONTRIBUTION

**Emily Charlesworth:** Conceptualization (equal); Data curation (lead); Formal analysis (lead); Investigation (equal); Methodology (equal); Project administration (lead); Visualization (equal); Writing – original draft (lead); Writing – review & editing (equal). **Alison Alderson:** Conceptualization (equal); Investigation (equal); Methodology (equal); Supervision (supporting); Visualization (equal); Writing – review & editing (equal). **Fiona Fylan:** Formal analysis (equal); Validation (equal); Writing – review & editing (equal). **Richard A Armstrong:** Formal analysis (equal); Validation (equal); Writing – review & editing (equal). **Aman Chandra:** Methodology (supporting); Project administration (supporting); Resources (supporting); Writing – review & editing (equal); Writing – review & editing (equal). **David Elliott:** Conceptualization (equal); Data curation (equal); Formal analysis (equal); Investigation (equal); Methodology (equal); Supervision (lead); Validation (equal); Visualization (equal); Writing – original draft (supporting); Writing – review & editing (equal).

## Supporting information

Figure S1

Figure S2

Table S1

Table S2

Table S3

Table S4

Table S5

## OPTOMETRIST SURVEY

### Practitioner profile

1. Approximately how long have you been qualified for?………………………………………………….....
2. Is the majority of your optometric work carried out as a Resident or Locum?
  - Resident
  - Locum
3. Where do you work?
  - Large multiple
  - Small multiple (less than 10 branches)
  - Independent
  - Hospital
  - University eye clinic
  - Other (please state) ………………………………………………………………………….......................

### Questionnaire Instructions

After consideration of a patient's history, signs, symptoms and results of all tests performed during the eye examination please tick the most appropriate decision that you would make in practice and briefly state your reason why. Part A begins on the next page.

### PART A

1. **Patient A**. Case history and clinical findings

A 65 year old retired teacher has bilateral cataracts which are impairing her vision. You have discussed referring her for bilateral surgery and she is happy to proceed. She is myopic and wears single vision distance glasses for everyday wear and takes her spectacles off to read.

**Table:**

RE −2.50 DS	VA 6/15+2	Add +2.50	N6
LE −2.50 DS	VA 6/15−2	Add +2.50	N8

At the point of referral would you discuss with Patient A their target refraction options?

(e.g. an emmetropic target refraction and distance spectacle independence, or a myopic target refraction and near spectacle independence, or an monovision target refraction and spectacle independence) and state your reasons why.

- Yes, discuss in person
- Yes, and state preference in referral letter
- No, leave the patient to discuss this with the Ophthalmologist / Hospital Eye Service

Reason for answer: ……………………………………………………………………………………………………………………………

1. **Patient B**. Case history and findings

A 72 year old retired builder has bilateral cataracts and he would like to be referred for bilateral cataract surgery. Primarily he wears monovision contact lenses with his right eye for distance vision and his left eye for near vision and likes his spectacle independence.

**Table:**

RE +1.25 DS	VA 6/15‐1	Add +2.50	N8
LE +2.25 DS	VA 6/15‐3	Add +2.50	N8

At the point of referral would you discuss with Patient B their target refraction options?

(E.g. an emmetropic target refraction and distance spectacle independence, or an myopic target refraction and near spectacle independence, or an monovision target refraction and spectacle independence) and state your reason why.

- Yes, discuss in person
- Yes, and state preference in referral letter
- No, leave the patient to discuss this with the Ophthalmologist/Hospital Eye Service

Reason for answer: ……………………………………………………………………………………………………………………………

### PART B

1. When do you think the patients target refraction should be discussed?
  - By the Optometrist at the point of referral (direct referral)
  - By the Optometrist at the point of referral (GP referral)
  - By the Ophthalmologist/Hospital eye service

## OPHTHALMOLOGIST QUESTIONNAIRE

### Practitioner Profile

4) Approximately how long have you been an Ophthalmologist for?

……………………………………………………………………………………………

5) Where do you work?

- NHS
- Private hospital
- Both

### Questionnaire Instructions

After consideration of a patient's history, signs, symptoms and results of all tests performed please tick the most appropriate decision that you would make in practice and briefly state your reason why. Part A begins on the next page.

## PART A

1. **Patient A**. Case history and clinical findings

A 65 year old retired teacher has bilateral cataracts which are impairing her vision. She has been referred her for bilateral surgery and she is happy to proceed. She is myopic and wears single vision distance glasses for everyday wear and takes her spectacles off to read.

**Table:**

RE −2.50 DS	VA 6/15+2	Add +2.50	N6
LE −2.50 DS	VA 6/15−2	Add +2.50	N8

Would you discuss with Patient A their target refraction options before surgery? Please state your reason why.

- Usually recommend an emmetropic target refraction
- Usually recommend a myopic target refraction
- Accept the patient's decision following a discussion of available options with the patient
- Accept the patient's decision based on the discussion they had with their optometrist
- No discussion

Reason for answer: ……………………………………………………………………………………………………………………………

1. **Patient B**. Case history and findings.

A 72 year old retired builder has bilateral cataracts and has been referred for bilateral cataract surgery. Primarily he wears monovision contact lenses with his right eye for distance vision and his left eye for near vision and likes his spectacle independence. The referral letter indicates he would prefer a monovision target refraction.

**Table:**

RE +1.25 DS	VA 6/15−1	Add +2.50	N8
LE +2.25 DS	VA 6/15−3	Add +2.50	N8

Would you consider a monovision target refraction for Patient B and state your reasons why.

- Yes, usually recommend a monovision target refraction
- No, usually recommend an emmetropic target refraction
- No, usually recommend a myopic target refraction
- Accept the patients decision following a discussion if available options with the patient

Reason for answer: ……………………………………………………………………………………………………………………………

## PART B

1. When do you think the patients target refraction should be discussed?
  - By the Ophthalmologist/Hospital eye service
  - By the Optometrist at the point of referral (direct referral)
  - By the Optometrist at the point of referral (GP referral)
2. Over the last year in what percentage of patients did you aim for an emmetropic target refraction?

Thank you for taking the time to complete this questionnaire. Please return in the freepost envelope provided.
