# Supplementary material for: Investigating target refraction advice provided to cataract surgery patients by UK optometrists and ophthalmologists
Source: Ophthalmic Physiol Opt. 2022 Feb 18;42(3):440–53. doi: 10.1111/opo.12957 (PMC9306962; doi:10.1111/opo.12957)

**Figure 1.** Venn diagram showing the themes found during qualitative analysis, and the percentage of comments found in each theme.

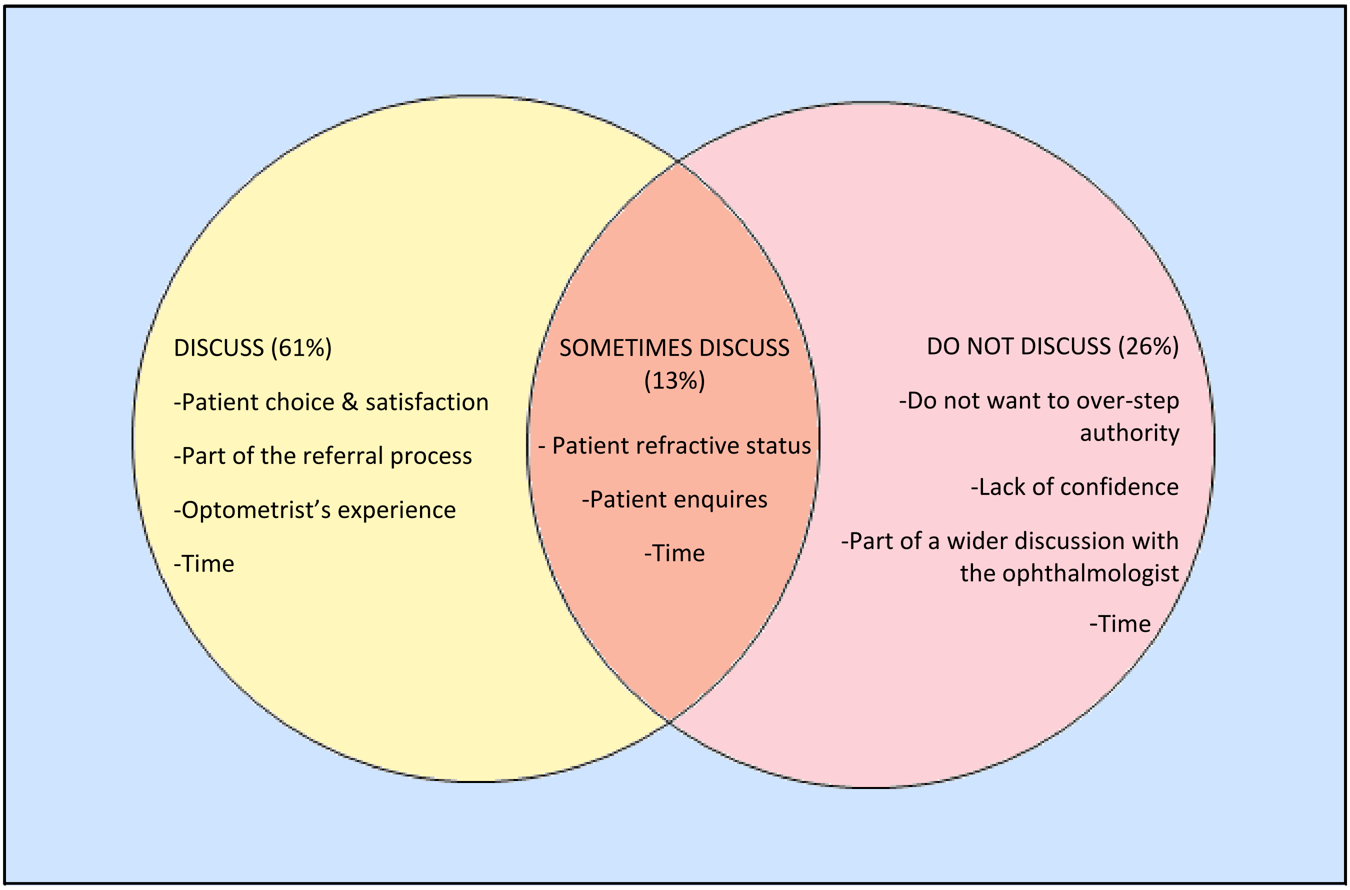

Supplement: Supplementary file 1 — Figure S1 [file OPO-42-440-s007.pdf]
