# Supplementary material for: Investigating target refraction advice provided to cataract surgery patients by UK optometrists and ophthalmologists
Source: Ophthalmic Physiol Opt. 2022 Feb 18;42(3):440–53. doi: 10.1111/opo.12957 (PMC9306962; doi:10.1111/opo.12957)
Supplement: Supplementary file 2 — Figure S2 [file OPO-42-440-s002.pdf]

Responses when optometrists and ophthalmologists were asked "When should target refraction first be discussed with the patient?"

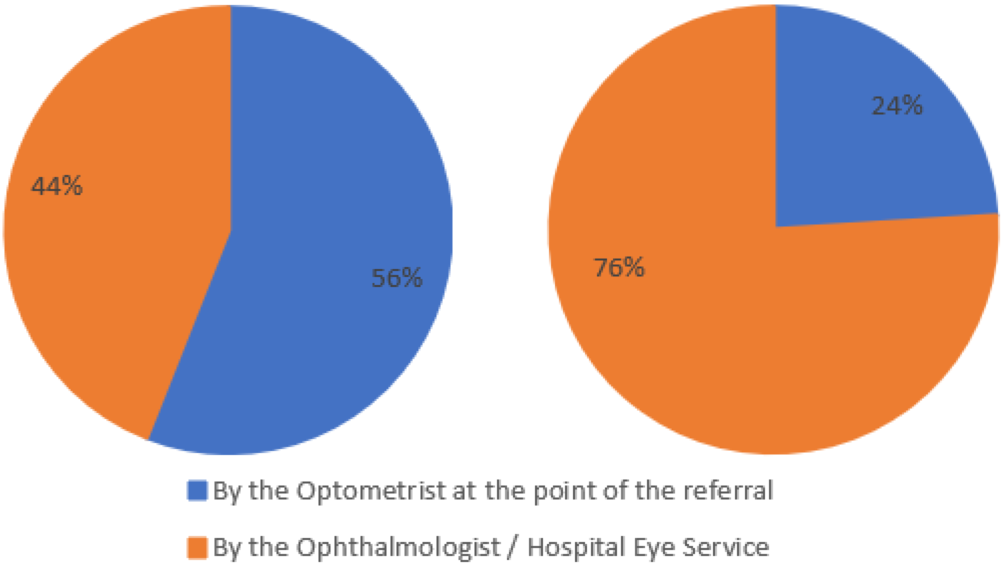

Figure 2: Showing the responses from optometrists (left) and ophthalmologists (right) when asked when target refraction should first be discussed with the patient.
