# Supplementary material for: Investigating target refraction advice provided to cataract surgery patients by UK optometrists and ophthalmologists
Source: Ophthalmic Physiol Opt. 2022 Feb 18;42(3):440–53. doi: 10.1111/opo.12957 (PMC9306962; doi:10.1111/opo.12957)
Supplement: Supplementary file 3 — Table S1 [file OPO-42-440-s005.docx]

| **Type of practice** | **% of total 437 questionnaires returned** | **Median (IQR) years qualified** |
| --- | --- | --- |
| Large Multiple | 195 (45%) | 10 (5-20) |
| Independent | 169 (39%) | 25 (17-35) |
| Hospital | 40 (9%) | 15 (8-22) |
| Small Multiple (less than 10 branches) | 18 (4%) | 21 (15-27) |
| University | 9 (2%) | 26 (8-30) |
| Domiciliary | 6 (1%) | 21 (15-29) |

**Table 1.**  The number of years qualified for 437 UK optometrists completing the survey divided by practice type.
