# Supplementary material for: Investigating target refraction advice provided to cataract surgery patients by UK optometrists and ophthalmologists
Source: Ophthalmic Physiol Opt. 2022 Feb 18;42(3):440–53. doi: 10.1111/opo.12957 (PMC9306962; doi:10.1111/opo.12957)
Supplement: Supplementary file 4 — Table S2 [file OPO-42-440-s003.docx]

| **Table 2.**  Survey responses for patient A subdivided into practice type. | **Yes discuss in person** | **Yes and state preference in referral letter** | **No leave the patient to discuss this with the ophthalmologist/HES** |
| --- | --- | --- | --- |
|  | n=149 | n=123 | n=165 |
|  | Median years qualified (IQR): 20 (10-30) | Median years qualified  (IQR): 22 (16-31) | Median years qualified  (IQR) 10 (15-20) |
| Large multiple  n=195 | 54 (28%) | 31 (16%) | 110 (56%) |
| Independent  n=169 | 72 (43%) | 63 (37%) | 34 (20%) |
| Small multiple  n=18 | 5 (28%) | 8 (44%) | 5 (18%) |
| Hospital  n=40 | 11 (28%) | 16 (40%) | 13 (33%) |
| Domiciliary  n=6 | 0 (0%) | 4 (67%) | 2 (33%) |
| University  n=9 | 7 (78%) | 1 (11%) | 1 (11%) |

**Table 2.**  Table showing the number of survey responses for patient A in each category. Each category is further subdivided into responses per practice type.
