# Supplementary material for: Investigating target refraction advice provided to cataract surgery patients by UK optometrists and ophthalmologists
Source: Ophthalmic Physiol Opt. 2022 Feb 18;42(3):440–53. doi: 10.1111/opo.12957 (PMC9306962; doi:10.1111/opo.12957)
Supplement: Supplementary file 5 — Table S3 [file OPO-42-440-s004.docx]

|  | **Yes discuss in person** | **Yes and state preference in referral letter** | **No leave the patient to discuss this with the ophthalmologist/HES** |
| --- | --- | --- | --- |
|  | n=113 | n=202 | n=122 |
|  | Median years qualified (IQR): 20 (10-30) | Median years qualified  (IQR): 20 (11-30) | Median years qualified  (IQR) 10 (5-20) |
| Large multiple  n=195 | 48 (25%) | 68 (35%) | 79 (40%) |
| Independent  n=169 | 49 (29%) | 94 (56%) | 26 (15%) |
| Small multiple  n=18 | 4 (22%) | 11 (61%) | 3 (17%) |
| Hospital  n=40 | 7 (28%) | 21 (53%) | 12 (30%) |
| Domiciliary  n=6 | 0 (0%) | 4 (67%) | 2 (33%) |
| University  n=9 | 5 (56%) | 4 (44%) | 0 (0%) |

**Table 3.**  Table showing the number of survey responses for patient B in each category. Each category is further subdivided into responses per practice type.
