# Supplementary material for: Investigating target refraction advice provided to cataract surgery patients by UK optometrists and ophthalmologists
Source: Ophthalmic Physiol Opt. 2022 Feb 18;42(3):440–53. doi: 10.1111/opo.12957 (PMC9306962; doi:10.1111/opo.12957)
Supplement: Supplementary file 6 — Table S4 [file OPO-42-440-s006.docx]

|  | n | Median (IQR) years qualified |
| --- | --- | --- |
| Discuss with both Patient A and B | 265 | 22 (13-31) |
| Do not discuss with Patient A or B | 114 | 10 (5-20) |
| Discuss with Patient B only | 50 | 15 (5-20) |
| Discuss with Patient A only | 8 | 14 (10-20) |

**Table 4.**  Optometrists’ median years qualified when subdivided into those that discussed target refraction with both patients, with neither patient, or with only one of the patients.
