# Supplementary material for: Investigating target refraction advice provided to cataract surgery patients by UK optometrists and ophthalmologists
Source: Ophthalmic Physiol Opt. 2022 Feb 18;42(3):440–53. doi: 10.1111/opo.12957 (PMC9306962; doi:10.1111/opo.12957)
Supplement: Supplementary file 7 — Table S5 [file OPO-42-440-s001.docx]

| Patient A  Would you discuss with Patient A their target refraction options before surgery? Please state your reason why. | % | Patient B  Would you consider a monovision target refraction for Patient B and state your reasons why. | % |
| --- | --- | --- | --- |
| Usually recommend an emmetropic target refraction | 24 | Yes, usually recommend a monovision target refraction | 52 |
| Usually recommend a myopic target refraction | 18 | No, usually recommend an emmetropic target refraction | 6 |
| Accept the patient’s decision following a discussion of available options with the patient | 52 | No, usually recommend a myopic target refraction | 2 |
| Accept the patient’s decision based on the discussion they had with their optometrist | 6 | Accept the patient’s decision following a discussion of available options with the patient | 40 |
| No discussion | 0 |  |  |

**Table 5.** Survey responses of 50 ophthalmologists for patients A and B.
